# Diet–Microbiota–Immune Interactions in Hepatocellular Carcinoma: An Immunometabolic and Spatial Perspective

**DOI:** 10.3390/nu18121911

**Published:** 2026-06-12

**Authors:** Asmaa E. Salem, Nourhan Nassar, Shimaa M. Emam, Shaimaa H. Negm, Wamidh H. Talib, Bence Raposa

**Affiliations:** 1 Institute of Emergency Care, Pedagogy of Health and Nursing Sciences, Faculty of Health Sciences, University of Pécs, 7621 Pécs, Hungary; 2 Department of Clinical Pathology, Faculty of Veterinary Medicine, Benha University, Moshtohor 13736, Egypt; nourhannassar@stu.ahau.edu.cn; 3 Department of Food Hygiene, Safety and Technology, Faculty of Veterinary Medicine, New Valley University, El-Kharga 72511, Egypt; shimaa.magdy@vet.nvu.edu.eg; 4 Home Economics Department, Faculty of Specific Education, Port Said University, Port Said 42512, Egypt; shaimaa_a_negm@yahoo.com; 5 Faculty of Allied Medical Sciences, Applied Science Private University, Amman 11937, Jordan; w_talib@asu.edu.jo; 6 Institute of Basics of Health Sciences, Midwifery and Health Visiting, Faculty of Health Sciences, University of Pécs, 7621 Pécs, Hungary

**Keywords:** hepatocellular carcinoma, gut microbiota, microbial metabolites, immunometabolism, gut–liver axis

## Abstract

Hepatocellular carcinoma (HCC) is the most frequent type of primary liver cancer and one of the leading causes of cancer-related mortality globally, with its incidence increasingly driven not only by viral hepatitis and alcohol-related etiologies but also by metabolic dysfunction-associated steatotic liver disease. Dietary intake can modify gut microbial activity and the production of microbial metabolites, which in turn may regulate hepatic immune signaling and metabolic pathways along the gut–liver axis. Microbiota-derived metabolites have emerged as important immunometabolic mediators linking dietary factors to hepatic immune responses and metabolic reprogramming. These metabolites, which have been shown to influence hepatic immune cell function and inflammatory signaling, include short-chain fatty acids, secondary bile acids, and tryptophan-derived indoles. Changes in the production and composition of these metabolites have been associated with immune dysregulation, chronic inflammation, and metabolic reprogramming that promote hepatocellular carcinoma development. This review highlights how diet–microbiota interactions reshape hepatic immunometabolism and discusses their potential translational relevance for prevention and therapeutic strategies in hepatocellular carcinoma.

## 1. Introduction

Hepatocellular carcinoma (HCC) stands out as the primary liver cancer with the highest incidence rate. According to global cancer statistics published in 2023, hepatocellular carcinoma (HCC) remains the third leading cause of cancer-related mortality worldwide, accounting for more than 800,000 deaths annually [1,2]. Among these deaths, viral hepatitis and alcoholism are still the leading causes. However, the increasing prevalence of metabolic dysfunction-associated steatotic liver disease (MASLD, previously NAFLD/NASH) has shifted the perspective of HCC’s origin toward a disease increasingly driven by metabolic and immune disorders [3,4].

The liver plays an important role in immunometabolic regulations, as it integrates different signals derived from diet, microorganisms, and the immune system. Its major physiological functions include nutrient processing, detoxification, and the establishment of immune tolerance to gut-derived antigens [5].

Long-term consumption of high-fat, high-fructose, or low-fiber diets has been associated with metabolic disturbances that promote oxidative stress, induce hepatocellular injury, and trigger inflammation, thereby altering hepatic immune programming through immunometabolic reprogramming [6].

Consequently, these alterations may contribute to shifts in macrophage polarization, T cell metabolism, and the expansion of myeloid-derived suppressor cells (MDSCs), creating tumor-supportive immune conditions [7].

Recent studies indicate that metabolic intermediates such as lactate, succinate, and fumarate can act as immunomodulatory signals that influence the HCC microenvironment through angiogenesis, macrophage activity, and T cell exhaustion [8].

Nutritional components, including polyunsaturated fatty acids, vitamins, and polyphenols, together with microbial metabolites such as short-chain fatty acids (SCFAs), bile acids, and tryptophan derivatives, have been reported to modulate key immune–metabolic pathways governed by AMPK, mTOR, PPAR, FXR, and AhR signaling [9,10]. Together, these interconnected processes form a diet-microbiota–liver axis that may influence hepatic inflammation, regeneration, and tumor evolution [11].

Microbial metabolites also directly influence tumor immunity and responsiveness to therapy. Bile acid derivatives engage FXR and TGR5 receptors to regulate antigen presentation and T cell recruitment, while microbial tryptophan catabolites activate the aryl hydrocarbon receptor (AhR) to modulate cytokine production and macrophage function [12,13].

Recent spatial omics studies further illustrate that distinct hepatic zones exhibit unique immunometabolic signatures, revealing region-specific effects of nutrient and microbial metabolites on immune remodeling [14]. Integration of spatial metabolomics, lipidomics, and imaging mass cytometry enables precise mapping of these interactions and provides new opportunities for precision immunometabolic interventions.

Previous reviews have examined metabolic or microbial aspects of HCC independently; however, few have integrated these perspectives into a unified immunometabolic framework. To address this gap, the present article adopts a narrative review approach, synthesizing mechanistic, translational, and clinical evidence linking diet, microbiota, and immune regulation in hepatocarcinogenesis. The literature was identified through searches of major scientific databases (PubMed, Science Direct). Priority was given to English-language publications directly relevant to the gut–liver axis, hepatic immune regulation, and spatial or multi-omics perspectives in HCC. In addition, the reference lists of key articles were screened to identify further relevant studies. No formal systematic review protocol or PRISMA-guided study selection process was applied.

**Figure 1:**
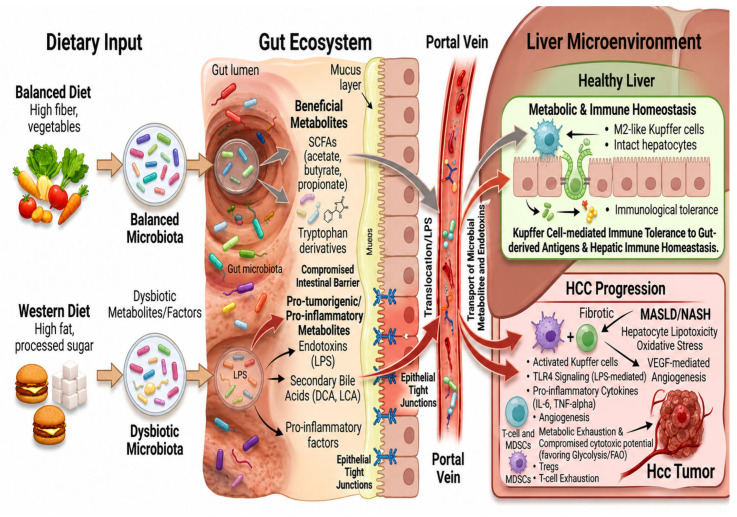
Conceptual overview of the Diet-Gut-Liver Axis in HCC. Dietary patterns influence gut microbial composition, metabolite production, intestinal barrier integrity, and hepatic immune homeostasis through the portal circulation. Beneficial microbial metabolites, including short-chain fatty acids (SCFAs) and tryptophan derivatives, may support immune regulation, whereas dysbiosis-associated metabolites such as lipopolysaccharide (LPS) and secondary bile acids may promote inflammatory and tumor-promoting signaling within the liver microenvironment. Abbreviations: SCFAs, short-chain fatty acids; LPS, lipopolysaccharide; DCA, deoxycholic acid; MASLD, metabolic dysfunction-associated steatotic liver disease. Grey arrows indicate beneficial or homeostatic interactions and metabolite transport, whereas red arrows indicate dysbiosis-associated, pro-inflammatory, or tumor-promoting pathways.

The review emphasizes how dietary components may shape microbial metabolism, how microbial metabolites can regulate hepatic immune signaling, and how these mechanisms may influence tumor progression or immune activation. This review aims to delineate the mechanistic, microbial, and nutritional pathways underlying hepatic immunometabolic remodeling and to propose translational strategies integrating dietary and microbiota-based modulation with immunotherapy in HCC, as shown in Figure 1. Particular emphasis is placed on the integration of immunometabolic signaling, microbial metabolite biology, spatial tumor microenvironment organization, and emerging precision nutrition approaches in hepatocellular carcinoma.

Much of the current mechanistic understanding derives from experimental and preclinical studies, whereas evidence from human HCC cohorts remains comparatively limited and is often associative in nature.

## 2. Nutritional Inputs Shaping the Gut–Liver Axis

The liver serves as a central metabolic and immune hub, integrating dietary and microbial signals to maintain systemic homeostasis. These diet–microbiota interactions are increasingly recognized as important modulators of hepatic inflammation and tumor-promoting immune environments in hepatocellular carcinoma. Nutritional inputs influence the gut–liver axis by modulating microbial diversity, metabolite production, and hepatic immune signaling. When this axis is disrupted by high-fat diets, refined sugars, or micronutrient deficiencies, oxidative stress and chronic inflammation emerge, leading to immune tolerance and an increased risk of hepatocellular carcinoma (HCC) [15,16] (Table 1).

The interaction of omega-3 fatty acids, dietary fibers, vitamins, and polyphenols strongly influences hepatic immune regulation through the gut microbiota and their metabolite synthesis. To be more specific, omega-3 fatty acids promote the proliferation of *Akkermansia* and *Lactobacillus* species, which in turn are responsible for the synthesis of resolvins and protectins. These lipid mediators inhibit IL-6 production and promote macrophage regulatory activity [15]. The fermentation process of dietary fibers is carried out by SCFA-producing bacteria, thus increasing the levels of butyrate and acetate that promote Treg differentiation and suppress NF-κB activation [9]. Vitamin D and selenium have similar effects on gut barrier function and redox balance, while polyphenols are reported to activate Nrf2 signaling and suppress inflammation by blocking NF-κB-mediated pathways [5,15,17].

### 2.1. Macronutrient Effects: Lipids, Proteins, and Carbohydrates

Macronutrients represent the primary modulators of microbial metabolism and immune responses along the gut–liver axis. High-fat diets are among the most significant dietary risk factors for HCC, largely due to their ability to induce gut dysbiosis, intestinal permeability, and hepatic lipid accumulation [6].

Excessive dietary lipids promote the expansion of bile-tolerant bacteria and reduce short-chain fatty acid (SCFA)-producing genera such as *Faecalibacterium* and *Bifidobacterium*, leading to increased lipopolysaccharide (LPS) translocation and activation of Toll-like receptor 4 (TLR4) signaling in Kupffer cells [19]. These immune shifts trigger chronic inflammation and upregulate tumor necrosis factor-alpha (TNF-α) and interleukin-6 (IL-6), both of which foster hepatocarcinogenesis [6].

Conversely, polyunsaturated fatty acids (PUFAs), particularly omega-3 fatty acids, exert hepatoprotective effects through the generation of anti-inflammatory lipid mediators such as resolvins and protectins, which regulate macrophage polarization and T cell activation [15].

Moreover, bile acid metabolism, closely tied to dietary fat intake, modulates the activity of nuclear receptors such as FXR and TGR5, influencing metabolic reprogramming and immune cell infiltration within the hepatic microenvironment [4].

Dietary proteins and their amino acid derivatives, including indole derivatives, polyamines, and tryptophan metabolites, are equally crucial in immunometabolic homeostasis. High-protein diets enriched with branched-chain amino acids (BCAAs) enhance hepatic mitochondrial oxidation and immune competence, whereas excessive red meat consumption, rich in heme iron and saturated fats, promotes reactive oxygen species (ROS) formation and inflammatory cytokine production [1].

Microbial fermentation of undigested proteins in the colon produces metabolites such as indole derivatives, ammonia, and hydrogen sulfide, some of which, like indole-3-propionic acid, have anti-inflammatory and epithelial-protective functions via aryl hydrocarbon receptor (AhR) activation [18].

Carbohydrate quality also shapes the hepatic immunometabolic axis. Diets high in refined carbohydrates increase hepatic lipogenesis and insulin resistance, whereas complex polysaccharides, such as inulin and β-glucans, enhance SCFA production by gut microbes, fostering T regulatory (Treg) cell development and reducing inflammation [9].

Butyrate, one of the most potent SCFAs, acts as a histone deacetylase (HDAC) inhibitor, regulating gene expression and promoting anti-inflammatory macrophage phenotypes [10].

### 2.2. Micronutrients and Bioactives: Vitamins and Polyphenols

Micronutrients (vitamins, trace elements, and plant-derived bioactives) are powerful regulators of both microbial ecology and hepatic immunity. Vitamin D, for example, exerts immunoregulatory effects by maintaining epithelial barrier integrity and suppressing pro-inflammatory cytokines through vitamin D receptor (VDR)-mediated transcriptional control. Low serum vitamin D levels correlate with altered gut microbiota composition and increased susceptibility to non-alcoholic steatohepatitis (NASH) and HCC [5].

Similarly, vitamin A influences innate immunity by modulating Kupffer cell differentiation and promoting gut IgA production, thereby shaping the hepatic immune microenvironment [16]. Vitamin E and selenium function as key antioxidants that mitigate lipid peroxidation and oxidative stress in hepatocytes. Selenium-containing enzymes such as glutathione peroxidase protect against ROS-induced DNA damage and modulate macrophage redox balance [15].

In parallel, polyphenols such as resveratrol, quercetin, and curcumin exert dual effects on microbial composition and hepatic metabolism. These compounds enhance the abundance of beneficial bacteria like *Akkermansia muciniphila* and *Lactobacillus*, leading to increased SCFA synthesis and decreased endotoxemia [17].

Polyphenols also activate the nuclear factor erythroid 2-related factor 2 (Nrf2) antioxidant pathway while inhibiting NF-κB-mediated inflammation signaling, effectively linking dietary bioactives with hepatic immunometabolic resilience [3].

Moreover, micronutrient deficiencies can exacerbate hepatic immune dysregulation. Vitamin B12 and folate deficiencies, for instance, disrupt methylation cycles and epigenetic regulation of immune-related genes, thereby altering macrophage and T cell responses [7]. Thus, balanced micronutrient intake not only prevents metabolic liver injury but also fortifies immune surveillance mechanisms critical for tumor suppression.

### 2.3. Nutrient Sensing and Signal Integration: AMPK, mTOR, PPAR, and SIRT1

At the molecular level, nutrient availability is sensed by highly conserved signaling pathways that regulate metabolism and immunity in hepatocytes and immune cells alike. The AMP-activated protein kinase (AMPK) pathway acts as a metabolic checkpoint linking energy stress to anti-inflammatory signaling. Activation of AMPK by dietary polyphenols or fasting suppresses hepatic lipogenesis, inhibits NF-κB, and promotes autophagy, processes that collectively hinder HCC progression [4].

Conversely, hyperactivation of the mechanistic target of rapamycin (mTOR) pathway, driven by nutrient excess, enhances anabolic metabolism and suppresses autophagic flux, creating a pro-tumorigenic environment [7].

Peroxisome proliferator-activated receptors (PPARs), particularly PPAR-α and PPAR-γ, serve as transcriptional regulators integrating lipid metabolism with immune function. Activation of PPAR-α by fatty acid derivatives enhances β-oxidation, reduces pro-inflammatory cytokine secretion, and modulates macrophage polarization toward an anti-inflammatory M2-like phenotype [11].

Likewise, SIRT1, a NAD+-dependent deacetylase, mediates the effects of caloric restriction and certain polyphenols, including resveratrol, on hepatic metabolism and immune function by deacetylating PGC-1α and NF-κB signaling components [18].

Collectively, these nutrient-sensing networks constitute an integrated signaling system that fine-tunes metabolic and immune balance in the liver. Dysregulation of these pathways through dietary excess, microbial imbalance, or micronutrient deficiency creates conditions conducive to immunosuppression and tumor growth. Thus, nutritional reprogramming targeting these molecular circuits may offer a viable therapeutic approach to restoring hepatic immunometabolic homeostasis.

## 3. Microbial Metabolites as Mediators of Nutritional Signaling

Microbial metabolites act as key biochemical messengers that translate dietary inputs into immune and metabolic responses along the gut–liver axis. Through fermentation, deconjugation, and biotransformation processes, gut microbes generate bioactive molecules capable of influencing hepatic metabolism, immune cell differentiation, and inflammatory signaling.

These metabolites, ranging from short-chain fatty acids (SCFAs) and bile acids to tryptophan catabolites and polyphenol derivatives, constitute the biochemical “language” of host–microbiota communication [19]. Their transport to the liver via the portal circulation enables direct modulation of hepatocytes, Kupffer cells, and hepatic stellate cells, shaping the immunometabolic landscape of hepatocellular carcinoma (HCC) [12]. Key microbial metabolites and their pathways are summarized in Table 2.

### 3.1. Short-Chain Fatty Acids (SCFAs)

SCFAs, primarily acetate, propionate, and butyrate, are the end-products of microbial fermentation of indigestible carbohydrates and dietary fibers. They represent a central link between diet and hepatic immune homeostasis. SCFAs regulate immune activity through binding to G-protein-coupled receptors (GPCRs) such as GPR41, GPR43, and GPR109A, as well as through epigenetic mechanisms involving histone deacetylase (HDAC) inhibition [9].

In experimental HCC models, butyrate and propionate have been shown to modulate hepatic inflammatory signaling and immune-cell differentiation, partly through effects on Kupffer-cell activation and FOXP3-associated regulatory T-cell responses [10]. Recent studies have revealed that SCFAs also modulate hepatic stellate cell (HSC) activation and fibrosis, key processes in HCC pathogenesis [20]. Butyrate-mediated AMPK activation downregulates collagen synthesis and limits extracellular matrix deposition, thereby attenuating fibrotic remodeling. Furthermore, SCFAs influence hepatocarcinogenesis through energy metabolic reprogramming, promoting oxidative phosphorylation in immune cells and enhancing anti-tumor immunity [9].

Conversely, dysbiosis resulting in decreased SCFA production is associated with metabolic dysfunction-associated steatotic liver disease (MASLD) and a higher risk of tumor-promoting inflammation [15].

### 3.2. Bile Acids (FXR/TGR5 Link)

Bile acids (BAs) are another major class of diet–microbiota co-metabolites with dual roles in metabolism and immunity. Beyond their classical function in lipid emulsification, BAs act as signaling molecules that interact with nuclear receptors such as the farnesoid X receptor (FXR) and membrane receptors such as Takeda G-protein-coupled receptor 5 (TGR5) [21].

Gut microbes catalyze bile salt hydrolase (BSH)-mediated deconjugation and dehydroxylation reactions, generating secondary bile acids such as deoxycholic acid (DCA) and lithocholic acid (LCA), which possess distinct immunomodulatory properties [12].

Activation of FXR in hepatocytes maintains lipid and glucose homeostasis while suppressing pro-inflammatory gene expression through small heterodimer partner (SHP)-dependent transcriptional regulation [5].

Conversely, TGR5 activation in Kupffer cells enhances cyclic AMP production, inhibiting TNF-α and IL-1β secretion and promoting an anti-inflammatory macrophage phenotype [11].

However, chronic exposure to secondary bile acids, particularly DCA, can induce oxidative stress, DNA damage, and senescence-associated secretory phenotypes in hepatic stellate cells, fostering a pro-tumorigenic microenvironment [14].

The immunomodulatory effects of bile acids are thus context-dependent: balanced bile acid pools sustain hepatic immune tolerance and metabolic homeostasis, while dysregulated microbial biotransformation of BAs skews FXR/TGR5 signaling toward chronic inflammation and oncogenesis. Recent metabolomic studies have demonstrated that dietary interventions targeting bile acid metabolism, such as prebiotic fiber or bile acid sequestrants, can normalize FXR signaling and inhibit HCC progression [19].

### 3.3. Tryptophan Metabolites and the AhR Pathway

Tryptophan metabolism represents a critical biochemical axis through which dietary amino acids and microbial activity converge to regulate immune responses. Gut microbes, including *Lactobacillus*, *Bacteroides*, and *Clostridium* species, convert tryptophan into indole derivatives such as indole-3-propionic acid (IPA), indole-3-aldehyde (IAld), and indole-3-acetic acid (IAA), which act as endogenous ligands of the aryl hydrocarbon receptor (AhR) [18]. AhR activation modulates transcriptional programs involved in xenobiotic metabolism, cytokine production, and immune tolerance.

In HCC, AhR signaling exerts both protective and pathogenic roles depending on the context of activation. Indole derivatives derived from microbial metabolism generally promote intestinal barrier integrity and reduce hepatic inflammation via IL-22 induction [16]. Conversely, kynurenine, a product of host tryptophan metabolism via indoleamine 2,3-dioxygenase (IDO1), drives T cell exhaustion and immune suppression when accumulated in the tumor microenvironment [4]. This metabolic competition for tryptophan between immune and tumor cells is emerging as a key determinant of HCC immunometabolic homeostasis.

Recent studies indicate that restoring microbial indole metabolism or inhibiting tumor-derived IDO1 activity can reactivate antitumor immunity and enhance the efficacy of immune checkpoint inhibitors [7]. Dietary tryptophan modulation, combined with probiotic supplementation of indole-producing bacteria, is thus being explored as a strategy to reprogram the hepatic immune microenvironment.

### 3.4. Polyphenol and Lipid-Derived Metabolites

Dietary polyphenols, such as flavonoids, stilbenes, and phenolic acids, undergo extensive microbial metabolism, generating low-molecular-weight metabolites that exert systemic immunometabolic effects. Gut bacteria such as *Eubacterium ramulus* and *Clostridium orbiscindens* biotransform polyphenols into compounds like urolithins, phenyl-γ-valerolactones, and hydroxyphenylpropionic acids [17].

These metabolites activate antioxidant and anti-inflammatory pathways in hepatocytes, notably through the nuclear factor erythroid 2-related factor 2 (Nrf2) axis, while suppressing NF-κB-mediated cytokine production [3].

In HCC models, polyphenol-derived metabolites enhance cytotoxic T lymphocyte activity and reduce tumor angiogenesis [6]. Moreover, resveratrol-derived microbial metabolites can upregulate SIRT1 and PGC-1α expression, thereby restoring mitochondrial function and metabolic flexibility in immune cells [10]. These findings support the concept of “nutritional immunotherapy,” in which dietary bioactives are leveraged to modulate microbial metabolism and immune signaling synergistically.

Lipid-derived metabolites, including conjugated linoleic acid (CLA), secondary bile lipids, and eicosanoids, further integrate dietary lipid metabolism with immune regulation. CLA, produced by certain *Lactobacillus* strains, has been shown to suppress HCC growth by activating PPAR-γ and inducing apoptosis in hepatocytes [21].

Additionally, microbial conversion of unsaturated fats can generate oxylipins that regulate macrophage polarization and T cell differentiation [14]. These multi-layered metabolic interactions illustrate the vast biochemical repertoire of the gut microbiota in modulating hepatic immunometabolic dynamics.

## 4. Conceptual Framework of Spatial Immunometabolism in Hepatocellular Carcinoma (HCC)

The concept of spatial immunometabolism in hepatocellular carcinoma (HCC) integrates tumor metabolic reprogramming with the functional states of immune cells within the tumor microenvironment. In this context, the tumor microenvironment (TME) plays a crucial role in modulating immune responses due to the metabolic alterations. Specifically, tumor cells in HCC often reprogram their metabolism by utilizing glycolysis and enhanced lipid biosynthesis, which supports rapid proliferation [8].

At the same time, immune cells, including T cells and macrophages, undergo metabolic changes in response to these alterations. As a result, the accumulation of lactate and hypoxia in the TME leads to impaired T-cell activity, thereby creating an immunosuppressive environment. Moreover, macrophages can adopt either a pro-inflammatory or immunosuppressive phenotype, depending on the metabolic pathways involved [12].

Furthermore, immune checkpoint inhibitors such as PD-1/PD-L1 blockade are key therapeutic strategies in HCC and may be influenced by the metabolic state of immune cells within the TME. Therefore, therapeutic approaches that integrate metabolic modulation with immune checkpoint represent promising strategies for improving treatment outcomes [6].

In addition, combination therapies that alter immune metabolism while enhancing immune checkpoint inhibition could be more effective. Overall, understanding the spatial organization of metabolic and immune interactions provides new insights into precision immunometabolic therapies in HCC [22,23].

## 5. Spatial Cellular and Immune Niches in Hepatocellular Carcinoma

Cirrhosis and HCC, together with contributing conditions such as metabolic dysfunction-associated steatotic liver disease (MASLD) and drug-induced liver injury (DILI), represent major causes of mortality related to liver disorders [2]. The liver, as a fundamental human organ, is responsible for the metabolism of nutrients and xenobiotics.

From an anatomical perspective, the liver contains functional units called liver lobules. Each lobule is histologically organized into a six-sided (hexagonal) arrangement of hepatocytes (parenchymal cells: PCs), forming a structured architecture that supports distinct metabolic and immunological activities.

The confluence of venous blood from the intestines with oxygenated arterial blood occurs at the portal vein level and flows through sinusoids toward the central vein [24]. These physiological gradients contribute to hepatic zonation, a spatial organization of hepatic functions that becomes significantly altered during chronic liver disease and hepatocellular carcinoma development.

### 5.1. Spatial Heterogeneity in Liver Tissue

Zonation is a phenomenon that involves the spatial distribution of hepatocyte functions along the liver sinusoid. As a central metabolic organ, the liver relies on zonation to perform multiple activities simultaneously, including glucose homeostasis and xenobiotic metabolism [25]. The liver is composed of diverse cell types, including hepatocytes organized into anatomical units known as lobules. These hexagonally structured lobules contain multiple layers of hepatocytes extending from the periportal to the pericentral regions. Gradients of oxygen, nutrients, and hormones are generated by blood flow from the portal veins and hepatic arteries at the corners of the lobules toward the central vein [26]. At the cellular level, hepatocyte spatial zonation is fundamental for regulating liver metabolic functions and maintaining metabolic homeostasis [27]. This spatial heterogeneity establishes the structural basis for regional differences in metabolism and immune activity within the liver.

Hepatic functions are therefore divided into multiple zones of intrahepatic metabolic activity and immune compartmentalization. Within this structured microenvironment, parenchymal hepatocytes, cholangiocytes, endothelial cells, and tissue-resident innate and adaptive immune cells form a dynamic cellular network. Their interactions are strongly influenced by spatial organization and localized signaling pathways [28]. During chronic liver disease, these spatially regulated processes become progressively disrupted, contributing to inflammatory remodeling and creating microenvironments that favor hepatocellular carcinoma initiation and progression.

### 5.2. Immune Cell Spatial Niches

**Figure 2:**
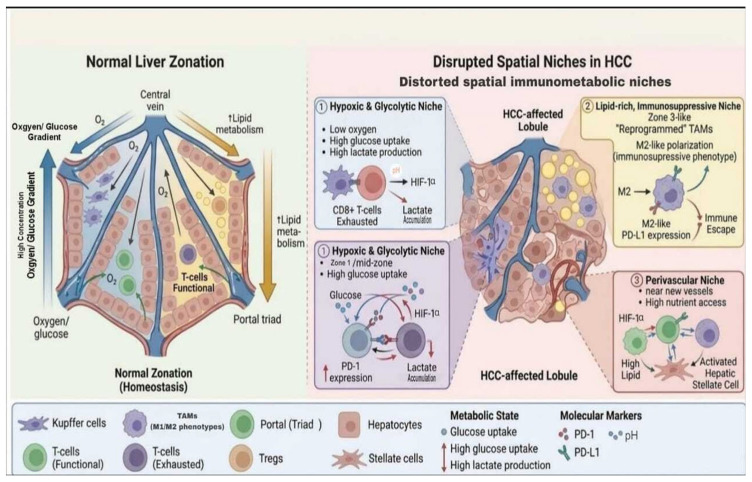
Conceptual diagram of spatial immunometabolism in hepatocellular carcinoma. The schematic compares normal liver zonation with disrupted spatial and metabolic niches observed in HCC, including hypoxic/glycolytic regions, lipid-rich immunosuppressive niches, and perivascular microenvironments. These spatial alterations are associated with lactate accumulation, PD-1/PD-L1 signaling, immune cell exhaustion, and tumor-promoting metabolic reprogramming. Abbreviations: TAMs, tumor-associated macrophages; PD-1, programmed cell death protein 1; PD-L1, programmed death-ligand 1; HIF-1α, hypoxia-inducible factor 1 alpha.

The specific characteristics of the structural and cellular organization of the liver define it as both a metabolic and an immunological organ, as shown in Figure 2. Remarkably, liver-resident macrophages, known as Kupffer cells, represent the predominant tissue-resident macrophage population in the human body. These cells are positioned along the sinusoidal lumen where they function as immunological sentinels responsible for clearing pathogens and cellular debris while maintaining immune tolerance under physiological conditions [27].

During acute or chronic liver injury, Kupffer cells may undergo functional impairment or cell death, accompanied by infiltration of circulating immune cells such as monocyte-derived macrophages. These recruited macrophages reshape the hepatic immune landscape and contribute to inflammatory and fibrotic responses associated with chronic liver disease and HCC progression [27].

Based on zonation, the liver can also be structurally characterized by the distribution of liver sinusoidal endothelial cells (LSECs), which play an important role during chronic liver injury and the early stages of fibrosis. Early molecular changes within specific zonal regions can influence disease progression, and fibrosis may potentially be counteracted if these alterations are detected before reaching advanced stages such as bridging fibrosis [24]. Consequently, this cellular and spatial organization contributes to the formation of specialized immune niches within the hepatic microenvironment.

In addition to hepatocytes and LSECs, several immune cell populations—including natural killer (NK) cells, invariant natural killer T (iNKT) cells, dendritic cells, and tissue-resident memory T cells—are localized to specific spatial niches within the liver parenchyma and sinusoids.

In contrast, recruited monocyte-derived macrophages and neutrophils accumulate preferentially in periportal or damaged regions, where they release inflammatory mediators and contribute to extracellular matrix remodeling during fibrosis [28,29].

The spatial proximity of immune cells to metabolically active hepatocytes allows them to sense local gradients of oxygen, glucose, and lipids. For example, pericentral zones enriched in glycolytic metabolism often promote immunosuppressive macrophage phenotypes due to local lactate accumulation and hypoxic conditions. In contrast, periportal regions characterized by oxidative metabolism tend to sustain more pro-inflammatory immune responses. Therefore, immune cell activity is closely linked to metabolic zonation, connecting local energy availability with immune surveillance and contributing to immunometabolism regulation during HCC development [26,28,29].

During chronic liver disease, the architecture of these immune niches becomes progressively distorted. Kupffer cells lose their steady-state distribution, while infiltrating macrophages and regulatory T cells accumulate in specific lobular regions. This spatial restructuring generates a pro-fibrotic and immunosuppressive microenvironment that promotes disease progression and contributes to hepatocellular carcinoma tumorigenesis [27,29,30].

### 5.3. Spatial Omics in Hepatocellular Carcinoma (HCC)

Spatial transcriptomics enables the quantification and spatial localization of gene expression within intact tissues while preserving tissue architecture and cellular context. Conventional bulk RNA sequencing averages gene expression across heterogeneous cell populations, which can obscure cellular heterogeneity. In contrast, single-cell RNA sequencing (scRNA-seq) provides high-resolution transcriptional profiling but disrupts spatial relationships during tissue dissociation [31,32]. Spatial omics approaches overcome these limitations by enabling the mapping of transcriptional programs directly onto histological structures within hepatocellular carcinoma tissues.

Through these technologies, spatial transcriptomics has revealed region-specific biological pathways and cellular interactions within the hepatocellular carcinoma tumor microenvironment. This spatial mapping highlights the organization of cellular heterogeneity and contributes to a deeper understanding of tumor biology and disease progression [33].

Single-cell RNA sequencing studies have significantly improved the understanding of cellular heterogeneity in hepatocellular carcinoma by identifying tumor cell subpopulations as well as diverse immune and stromal compartments [34,35]. However, the tissue dissociation required for single-cell analysis disrupts spatial cell–cell interactions, which limits the interpretation of spatially organized cellular networks [36]. Spatial transcriptomics complements these approaches by restoring anatomical context, allowing the identification of immune niches and functionally distinct tumor regions within HCC tissues [37,38].

Spatial multi-omics analyses have further shown that tumor cells and stromal components, particularly cancer-associated fibroblasts (CAFs), represent key elements of the HCC microenvironment and exhibit distinct spatial organization. CAFs promote tumor growth and metastasis through cytokine secretion and extracellular matrix remodeling.

At the same time, they contribute to immune evasion by recruiting monocytes and dendritic cells and inducing immunosuppressive phenotypes [39]. Spatial transcriptomic studies have demonstrated that tumor cells and CAFs frequently colocalize within HCC tissues, facilitating intercellular communication and paracrine signaling [40].

Integrated spatial transcriptomic and single-cell analyses have also uncovered key ligand–receptor interactions that mediate tumor–stromal communication in hepatocellular carcinoma [38].

One example is the interaction between tumor-derived secreted phosphoprotein 1 (SPP1) and the CD44 receptor expressed on hepatic stellate cells. This signaling interaction promotes the activation and differentiation of stellate cells into CAFs. Disruption of this signaling axis has been associated with reduced CAF infiltration and enhanced T-cell cytotoxic activity, highlighting the functional importance of spatially resolved molecular interactions within the immunosuppressive tumor microenvironment and their influence on responses to immune checkpoint blockade [40].

Beyond stromal interactions, spatial transcriptomics has also revealed distinct immune niches within hepatocellular carcinoma tissues, including immune-enriched and immune-excluded regions [41]. These spatial immune patterns are closely associated with immunotherapy outcomes. Tumors characterized by increased CD8^+^ T-cell infiltration and clonal expansion tend to show improved responses to immune checkpoint inhibitors, whereas stromal barriers that prevent T-cell infiltration are often associated with therapeutic resistance [42]. These findings highlight the translational potential of spatial omics approaches for predicting treatment responses and improving precision immunotherapy strategies in hepatocellular carcinoma, as shown in Figure 2.

## 6. Mechanistic Pathways Connecting Diet-Derived Metabolites and Spatial Immunity

Immune cell activity, activation, and differentiation are all heavily influenced by cellular metabolism. Different immune cell subsets require distinct metabolic programs to fulfill their bioenergetic and biosynthetic demands. It is evident that immunological function and the body’s reaction to illness are significantly impacted by nutrition [43].

In addition to helping with lipid absorption, bile acids also function as signaling molecules by interacting with different receptors. Bile acid metabolism is an essential regulator of intestinal homeostasis because bile acids are constantly recycled through the enterohepatic circulation and are biotransformed by gut bacteria. Two important bile acid receptors, the farnesoid X receptor (FXR) and G protein-coupled bile acid receptor 1 (TGR5), are highly expressed in the intestinal epithelium and are essential for preserving bile acid homeostasis and intestinal barrier function [44].

### 6.1. FXR and TGR5

The first bile acid receptor to be discovered was the nuclear receptor FXR [45], which is mostly expressed in organs involved in the hepatic-intestinal circulation of bile acids. In HCC, dysregulated FXR/TGR5 signaling has been associated with chronic inflammation, altered bile acid homeostasis, immune evasion, and metabolic adaptation within the tumor microenvironment [42,46].

Although the activation capacity of FXR differs among BAs, several bile acids can activate FXR signaling pathways. Hydrophilic bile acids activate FXR in the following order: DCA > CA = CDCA > LCA [47], whereas hydrophilic bile acids such as UDCA do not activate FXR, and muricholic acids (MCA) antagonize FXR [48].

BAs stimulate FXR in liver cells, thereby regulating bile acid metabolism by modifying genes linked to bile acid secretion [42]. Additionally, liver disease can be improved by FXR activation. Treatment with the FXR agonist obeticholic acid (OCA) produced a number of associated liver effects in patients with non-alcoholic fatty liver disease (NAFLD) or in mouse models, including lowering inflammation and triglycerides (TAGs), as well as reducing steatohepatitis and liver fibrosis [49]. IBABP has a strong affinity for BAs, and FXR also affects molecules involved in bile acid transport. FXR modulates the IBABP gene in intestinal epithelial cells to maintain bile acid homeostasis [50].

TGR5 is an important membrane bile acid receptor belonging to the G protein-coupled receptor family [51,52]. TGR5 regulates bile secretion, microcirculation, inflammation, regeneration, and gallbladder filling in the liver [53].

Additionally, TGR5 acts as a negative regulator of inflammation. In mouse models, TGR5 deficiency increases susceptibility to liver injury following lipopolysaccharide administration, resulting in elevated inflammatory cytokine levels and increased hepatocyte death [54].

The hierarchy of BAs’ ability to activate TGR5 is as follows: LCA > DCA > CDCA > CA [55].

When bile acids bind to TGR5, a complex composed of G proteins (αs, β, and γ) is released. Activation of TGR5 stimulates insulin production in pancreatic beta cells via the G-αs/cAMP/Ca^2+^ signaling pathway [56]. TGR5 is also essential for maintaining intestinal barrier function; bile acids activate TGR5 in intestinal epithelial cells, which enhances barrier integrity through stimulation of myosin light chain kinase (MLCK) signaling. Furthermore, TGR5 regulates the expression of cholesterol 12α-hydroxylase (CYP8B1), a key enzyme involved in bile acid synthesis, thereby reducing the proportion of 12α-hydroxy bile acids in the bile acid pool [57].

### 6.2. AHR Pathway

The aryl hydrocarbon receptor (AhR) is a widely expressed, ligand-activated transcription factor belonging to the basic helix-loop-helix/Per-Arnt-Sim (bHLH/PAS) superfamily of sensors of endogenous and foreign signals. It was first discovered to be involved in the metabolism of xenobiotics, especially those that contain aromatic hydrocarbons [58].

AhR performs crucial roles in preserving homeostasis [59]. Within the HCC microenvironment, AhR signaling has been linked to microbiota-dependent immune regulation, altered T-cell activity, and immunosuppressive metabolic adaptation. When a ligand binds to AhR in the cytoplasm, it moves to the nucleus and combines with AhR nuclear translocator (ARNT) to form a complex [60]. Following ligand binding, AhR regulates transcriptional programs involved in immune and metabolic signaling [61].

AhR is the only member of this superfamily that is known to bind naturally occurring xenobiotics, in contrast to other bHLH/PAS proteins [59].

Human disorders, including cancer, autoimmune diseases, inflammatory disorders, and metabolic diseases, have been linked to mutations in the AhR gene or close to AhR target genes, mostly through pathways including altered xenobiotic metabolism, disrupted gene regulation, and compromised immune function [62,63,64].

Following ligand binding and heterodimerization with ARNT, AhR regulates gene expression by binding to XRE sequences, thereby influencing multiple biochemical pathways involved in diverse biological processes [31].

### 6.3. Nrf2/NF-κB Axis

Under basal conditions, Nrf2 is the primary regulator of redox homeostasis and is strictly controlled by Keap. Highly reactive cysteine residues found in Keap1are essential for its functional sensitivity to stress [65].

Upon oxidative or electrophilic stress, these residues undergo modifications that disrupt Nrf2 repression and promote its stabilization [66]. Nrf2 is essential for preserving mitochondrial integrity, enhancing energy generation, and averting oxidative damage linked to liver cancer. In HCC, persistent activation of Nrf2/NF-κB signaling may contribute to oxidative stress adaptation, inflammatory remodeling, and resistance to antitumor immune responses within the tumor microenvironment [67].

### 6.4. AMPK/mTOR Signaling

The anabolic and catabolic pathways that govern cell growth, proliferation, and survival are shaped by AMPK and mTORCs, which play crucial roles in regulating cell metabolism in many cancer types. A crucial energy sensor, AMPK, is triggered when cellular energy levels are low, whereas mTOR’s activity is seen when nutrition levels are high [68]. In hepatocellular carcinoma, dysregulated AMPK/mTOR signaling is closely associated with metabolic reprogramming, mitochondrial dysfunction, and impaired antitumor immune-cell activity. AMPK represents a key link between the immune microenvironment and tumor cell energy metabolism [69]. According to recent research, AMPK demonstrates anticancer immunological activity by interacting with prominent elements in the cancer microenvironment and influencing the functions of macrophages, myeloid suppressor cells, and T lymphocytes [70]. Moreover, AMPK can prevent the synthesis of cytokines and chemokines [71].

**Figure 3:**
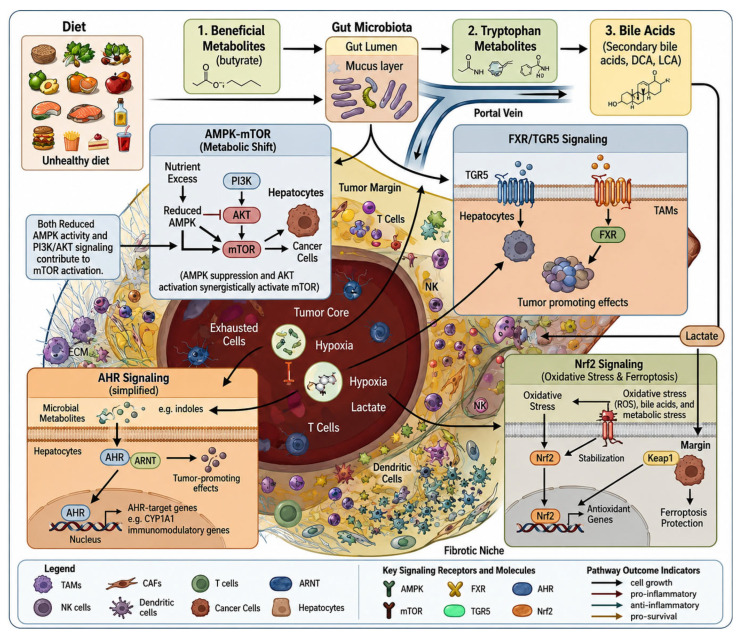
Key signaling pathways (e.g., AMPK–mTOR, FXR/TGR5, AhR, Nrf2) in the hepatic tumor microenvironment. The schematic illustrates how gut microbiota-derived metabolites influence major signaling pathways, including AMPK–mTOR, FXR/TGR5, AhR, and Nrf2 signaling, thereby modulating oxidative stress, immune cell activity, inflammatory responses, and tumor-promoting metabolic reprogramming. Abbreviations: AhR, aryl hydrocarbon receptor; FXR, farnesoid X receptor; TGR5, Takeda G protein-coupled receptor 5; AMPK, AMP-activated protein kinase; mTOR, mechanistic target of rapamycin; Nrf2, nuclear factor erythroid 2-related factor 2. Arrow colors indicate different biological outcomes: black arrows represent signaling or molecular interactions, red/brown arrows indicate pro-inflammatory or tumor-promoting effects, blue/green arrows indicate anti-inflammatory effects, and T-shaped lines indicate inhibitory interactions.

Furthermore, the control of the tumor microenvironment is greatly aided by the mTOR signaling pathway [72]. The development, differentiation, survival, and operation of both innate and adaptive immune cells are regulated by the mTOR cascade [73]. Additionally, mTOR signaling modulates inflammatory responses and immune cell activity in the tumor microenvironment, thereby influencing tumor progression and antitumor immunity [74,75,76]. Additionally, it has recently been demonstrated that AMPK and mTOR have a role in mitochondrial dynamics in the development of liver cancer [77], as shown in Figure 3.

### 6.5. Epigenetic and Redox Interactions

Additional epigenetic mechanisms, including DNA methylation, histone methylation, chromatin remodeling, and non-coding RNA regulation, have also been implicated in hepatocarcinogenesis and immune dysregulation within the tumor microenvironment. These processes may alter the expression of tumor suppressor genes, inflammatory mediators, and metabolic regulators involved in HCC progression [78]. Importantly, oxidative stress (ROS) is a primary contributor to these epigenetic modifications, demonstrating a link between redox imbalance and the emergence of cancer [79]. Metabolic reprogramming frequently alters the expression of epigenetic modifiers in the liver [80], and targeting these alterations may enhance therapeutic responses in liver cancer [81]. Targeting the synthesis of metabolites required for epigenetic modification, in addition to direct epigenetic therapy, may be a potential strategy for liver cancer. Hepatitis and HCC are caused by a drop in S-adenosyl-L-methionine (SAM) or a change in the SAM-producing enzyme MAT1A to MAT2A, which also encourages the spread of HCC tumor cells [82]. Additionally, histone acetylation in HCC cells is connected with intracellular acetyl-CoA levels [83]. By preventing acetyl-CoA carboxylation, acetyl-CoA carboxylase inhibitors can reduce lipogenesis in HCC tumor cells. Because acetyl-CoA serves as a substrate for histone acetylation, its regulation can influence gene expression and chromatin remodeling [84].

Furthermore, it has been observed that ACSS2 inhibits the metastasis of HCC tumor cells by acetylating HIF-2α [85]. Another enzyme involved in acetyl-CoA metabolism and histone acetylation is acetyl-CoA thioesterase 12 (ACOT12). However, Lu et al. showed that HCC cells’ downregulation of *ACOT12* raised intracellular acetyl-CoA levels, which triggered *TWIST2* expression to encourage tumor cell metastasis [83]. Additionally, by acetylating H3K9Ac at the interferon promoter in vitro, the supplement of acetate and acetyl-CoA can functionally reverse the autophagy of tumor-infiltrating T cells and increase the production of IFN-γ [86]. One significant metabolic stressor that regulates the proliferation of tumor cells is alterations in the redox state. Reactive oxygen species (ROS) buildup is typically the source of a redox imbalance [87]. Increased tumor cell motility and proliferation, genomic instability, mitochondrial malfunction, and activated tumor-promoted signaling pathways can all result from even a slight rise in ROS [88]. Overexposure to ROS causes permanent cell death, including ferroptosis and apoptosis [89]. Research has demonstrated that epigenetic regulation and redox imbalance, both separately and together, encourage carcinogenesis [90,91].

ROS are primarily generated through one-electron transfer reactions to molecular oxygen, producing species such as superoxide, hydrogen peroxide, and hydroxyl radicals [92]. The mitochondrial electron transport chain, endoplasmic reticulum (ER) stress, and nicotinamide adenine dinucleotide phosphate (NADPH) oxidases (NOXs) are the primary sources of ROS [93]. Studies have shown that ROS has a dual-edged effect in promoting cancer and preventing liver cancer [94].

## 7. Nutritional and Microbiota-Based Interventions to Reprogram the HCC Immunometabolome

Nutritional and microbiota-based therapies are an exciting approach to overturn the hepatic immunometabolome prior to malignant transformation and tumor immune escape. These interventions have been demonstrated to complement cytotoxic therapies because they remodel the gut microbiota and its metabolites, and restructure immune cell interactions in the tumor microenvironment [95]. The hallmark immunological characteristics of HCC are dendritic cell dysfunction, dysfunction of the natural killer (NK) cytotoxicity, T-cell exhaustion, and the presence of immunosuppressive cell populations, including regulatory T cells and myeloid-derived suppressor cells, which stimulate tumor growth and inhibit effective antitumor immunity [96].

There is growing evidence that the gut microbiota has a central role to play in the regulation of the hepatic immune landscape, both by metabolic and immunomodulatory mechanisms. Microbial-derived metabolites, such as SCFAs, secondary bile acids, and tryptophan metabolites, have the ability to modulate immune cell differentiation, activation, and metabolic programming in the liver [97].

Therefore, a rational and innovative way to reprogram the HCC immunometabolome and enhance immune-mediated tumor control is the application of targeted nutritional interventions and microbiota-based interventions.

### 7.1. Probiotics

Probiotics and prebiotics are a complementary approach to controlling the gut microbiota and its metabolic products [98], as in chronic liver disease and HCC. These treatments normalize the gut microbiota, restore the functionality of the gut barrier, and recalibrate the major immune and metabolic signals. When combined, they reorganize the immune condition of the liver to more efficiently recognize and attack tumors. Probiotic supplements containing *Lactobacillus*, *Bifidobacterium*, *Akkermansia muciniphila*, and selected *Clostridia* species have been shown to have consistently antitumor activity in preclinical models of HCC [99,100].

One of the key physiological mechanisms is the increase in the intestinal barrier functionality, leading to a reduction in the gut permeability and preventing the passage of LPS into the liver via the portal vein. Probiotics attenuate LPS-induced Toll-like receptor 4 (TLR4) signaling in Kupffer cells and inhibit chronic hepatic inflammation, a major cause of fibrosis progression and hepatocarcinogenesis [101,102].

Probiotics on an immunometabolic scale also raise SCFA production, primarily butyrate and propionate, which have a potent impact on immunometabolism behavior and the activity of liver immune cells. These metabolites act as histone deacetylase (HDAC) and AMP-activated protein kinase (AMPK) inhibitors and activators of a metabolic reprogram of macrophages [103].

The change predisposes an anti-inflammatory macrophage phenotype and breaks pro-tumorigenic inflammatory signaling loops in the liver. The adaptive antitumor immunity is also boosted directly by SCFAs [104].

It has also been reported that butyrate promotes the expression of chemokines, including CXCL11, which facilitates the recruitment of cytotoxic immune cells, such as natural killer cells, into HCC tissues.

Simultaneously, acetate may suppress immunosuppressive signals by suppressing IL-17A secretion by Type 3 innate lymphoid cells (ILC3s), which alters the cytotoxic T-cell infiltration in the presence of PD-1 blockade. All these effects are mediated by metabolic reprogramming and the action of histone deacetylase (HDAC), which improves the effector activity of CD8^+^ T-cells and plays a role in remodeling immunosuppressive cell populations within and around the tumor [97,99,105].

### 7.2. Prebiotics

Prebiotics enhance the protective effects of the microbiome by enhancing the populations of SCFA-producing bacteria by serving as food to them [106]. Prebiotic diets like inulin, fructo-oligosaccharides, galacto-oligosaccharides, and resistant starch augment the generation of acetate, propionate, and butyrate, thus maintaining the availability of microbial metabolites.

Prebiotic fermentation results in SCFAs that improve the effectiveness of the epithelial barrier and alter mucosal immune homeostasis, which in turn decreases microbial translocation and systemic inflammation, leading to the development of cirrhosis and HCC [107].

Butyrate presents special antitumor action at the cellular level. Tumor cells have defective mitochondrial use of butyrate as compared to normal epithelial cells, and it accumulates in the nucleus, where it inhibits HDAC activity and disturbs transcriptional programs that prevent proliferation and tumorigenesis [108].

The increased levels of SCFAs by the portal vein in the liver aid the maturation of dendritic cells and antigen presentation, which ensure the restoration of effective activation of T-cells and the elimination of immune exhaustion in the HCC microenvironment. Even though SCFAs are able to sustain regulatory T cells, in the inflamed HCC context, they paradoxically augment antitumor immunity by augmenting effector T-cell metabolic efficiency [109].

There is emerging clinical evidence of the translational utility of probiotic- and prebiotic-based interventions in HCC. Probiotics and prebiotics are found to increase the microbiota diversity of the gut, lipid metabolism, inflammation, and immunity of HCC patients, and early evidence of dietary interventions has been associated with increasing progression-free survival [110]. Taken together, these results suggest that probiotics and prebiotics are prospective adjunctive interventions that can reprogram the HCC immunometabolome to improve immune-mediated tumor control.

### 7.3. Functional Foods and Phytochemicals

The immunometabolic effects of functional foods and phytochemicals are also associated with microbial biotransformation into bioactive metabolites to a significant degree [111]. Foods rich in polyphenols are not well absorbed in the small intestine. As a result, they arrive at the colon, where they are metabolized to produce phenolic acids and urolithins, which possess potent anti-inflammatory and anticancer properties [112,113]. Microbiota polyphenol metabolites activate Nrf2, which boosts antioxidant defenses and inhibits ROS-mediated NF-kB communication in hepatocytes and immune cells [114].

The effect of this redox reprogramming is the attenuation of DNA damage and suppression of inflammatory macrophage activation. Moreover, a series of indole-based polyphenol metabolites can interact with the AhR, inducing the generation of IL-22 and epithelial integrity and thus indirectly preventing chronic hepatic inflammation [115].

Another group of functional nutrients that have immunometabolic potential is the omega-3 polyunsaturated fatty acids (PUFAs). The metabolism of omega-3 PUFAs by gut microbes produces specialized pro-resolving mediators (SPMs) that actively end inflammation, inhibit angiogenesis, and increase the cytotoxicity of CD8^+^ T-cells [116,117].

The omega-3 fatty acid-derived metabolites in HCC models inhibit COX-2/PGE2 signaling and change the immune cells towards fatty acid oxidation, a metabolic state that is associated with longer-lasting antitumor immune responses [118]. Fermented foods, when consumed regularly, promote an increase in microbial diversity and select bacteria that alter bile acid metabolism to produce bile acid signaling that decreases liver inflammation and cancer-related pathways [12].

### 7.4. Diet Composition Interventions

Mediterranean-style dietary patterns are high in dietary fiber, monounsaturated fats, polyphenols, and omega-3 polyunsaturated fatty acids. These dietary components facilitate healthy changes in the gut microbiota, increase production of SCFAs, normalize bile acid signaling, and decrease systemic inflammation [119].

They promote anabolic metabolism of tumor cells through inhibition of mTOR at the immunometabolic level in tumor cells and maintenance of immune cell metabolic integrity, thus lowering HCC risk and delaying disease progression [120]. In the same manner, gut microbiota responses to low-fat, high-fiber diets result in increased *Prevotella* and *Bacteroides* diversity by decreasing Firmicutes, promoting anti-inflammatory and antitumor metabolic conditions [121].

Along with dietary composition, energy intake is also a strong regulator of immunometabolic signaling. Caloric restriction and intermittent fasting have become powerful immunometabolic interventions. The strategies lead to AMPK activation, block mTOR signaling, and autophagy, which enhances immune cell resistance to stress and curbs the proliferation of myeloid-derived suppressor cells (MDSCs) in HCC models [122]. The gut microbial changes induced by fasting enhance SCFAs as well as ketone body generation, which supplements other metabolic powers of antitumor immunity [110]. All these findings are indicative of the fact that dietary control of the gut microbiota is a promising and mechanistically sound approach to boost immune vigilance and therapeutic responses in HCC.

### 7.5. Personalized Nutrition Strategies

The inter-individual differences in microbiota composition of the gut, etiology of liver disease, and tumor spatial architecture highlight the importance of individual-based nutritional approaches in HCC [123]. The discovery of microbiome sequencing, metabolomics, and spatial immune profiling can now identify patient-specific immunometabolic vulnerabilities, offering a rationalized foundation of dietary interventions to individual disease settings [124].

As an example, customized prebiotic or synbiotic interventions might be of the greatest benefit to those individuals who have low production of SCFAs, whereas patients with disturbed bile acid metabolism might react better to diets aimed at regulating FXR and TGR5 signaling. Notably, the immune checkpoint inhibitors can be integrated with such nutritional interventions to induce immune metabolic function, address immunosuppressive limitations in tumor microenvironments, and potentially increase tumor response to therapy [125,126].

Integration of microbiome, metabolic, and immune profiling may facilitate the development of personalized nutritional strategies aimed either at disease-risk reduction and prevention in high-risk populations or at supportive metabolic modulation in patients with established HCC. Representative preclinical studies investigating nutritional and microbiota-based modulation in experimental HCC models are summarized in Table 3.

## 8. Emerging Technologies to Map and Manipulate the Nutritional Immunometabolome

### 8.1. Spatial Metabolomics and Lipidomics

Metabolomics and lipidomics provide powerful tools for characterizing metabolic alterations within the tumor microenvironment and identifying metabolite–immune interactions in HCC.

### 8.2. Imaging Mass Cytometry

Imaging Mass Cytometry (IMC) integrates proteomics with spatial imaging, enabling the simultaneous quantification of over 40 markers using metal-tagged antibodies and time-of-flight mass spectrometry. In HCC, IMC has been instrumental in delineating immunometabolic circuits, the co-localization of exhausted CD8^+^ T cells with PD-L1^+^ myeloid cells in regions of high lactate accumulation [128]. This spatially resolved approach provides an unparalleled ability to correlate immune phenotypes with metabolic stressors within the tumor architecture.

Recent IMC studies have combined metabolic enzyme labeling glutaminase, hexokinase II, and IDO1 with immune cell phenotyping to reconstruct a spatial atlas of immunometabolic interactions in HCC [129]. These data have revealed that metabolic heterogeneity, particularly in perivascular niches, is closely tied to immune exclusion and angiogenic remodeling. Moreover, spatial models have demonstrated that SIRT3^+^ macrophages cluster near necrotic zones, implying adaptive shifts in mitochondrial metabolism that promote local immunosuppression [21].

Emerging workflows combine IMC with MSI-based metabolomics to co-register molecular and cellular data, capturing both metabolite localization and immune signaling simultaneously [130]. This combinatorial mapping helps to pinpoint metabolic vulnerabilities in immunosuppressive microenvironments and supports the rational design of therapies that reprogram immune metabolism in situ.

### 8.3. Multi-Omics Integration

Throughout the past decades, advances in high-throughput technologies and the decreased cost of large-scale data production have mainly changed biomedical research by allowing the simultaneous enhancement of different molecular layers [131]. Merging these heterogeneous datasets offers a systems-level perspective on biological pathways and enables the examination of complex molecular interactions [131]. Despite the fact that individual omics approaches were central to medical research, their integration provided a fuller comprehension of disease biology [132,133]. Likewise, combining epigenomic and transcriptomic data provides deeper insight into regulatory mechanisms [123]. Under sequential and parallel strategies, computational approaches for multi-omics integration are commonly categorized, based on how data from multiple omics layers are linked [124]. The application of these strategies to disease subtyping, patient stratification, and biomarker discovery highlights the growing significance of multi-omics integration [125,126,128].

### 8.4. Computational Modeling and Artificial Intelligence

The complex interplay of diet, the gut microbiome, microbial metabolites, host metabolism, and tumor immunity in HCC is beyond the explanatory ability of classic statistical methods. Therefore, computational modeling and AI have become the key to integrating high-dimensional data, decoding immunometabolic crosstalk, and creating predictive frameworks [129].

Mechanistic computational models, especially constraint-based metabolic models, can be used to model the reorganization of microbial metabolism and consequent host responses to dietary inputs [130]. GEMs the functional connections between microbial metabolic activity and disease state, and how microbial functions, as opposed to taxonomy, can stratify individuals and forecast metabolite profiles that can be applied to immune regulation [131].

In relation to HCC, these models offer a platform on which the in silico outcomes of how dietary interventions can change SCFAs, bile acids, and other immunomodulatory metabolites can be predicted. Beyond mechanistic modeling, integrative computational models that couple multi-omics data, such as metagenomics, metabolomics, and transcriptomics data with clinical phenotypes, have been useful in the process of identifying biomarkers and functional pathways in relation to HCC progression [132].

Complementary machine learning and AI models allow combining dietary, microbiome, genomic, and clinical data to obtain future insights into interactions between diet, microbiome, and risk of disease. Using AI includes the digital gut twin, which uses nutrient databases, host and microbial multi-omics profiles to provide a roadmap [134]. Recent innovations in spatially resolved omics technologies further expand AI applications. Spatial transcriptomics and proteomics can map immune and metabolic states in intact tissue architecture [135].

Overall, computational modeling and AI offer a balance of nutritional immunotherapy on a personalized basis in HCC [136]. AI can be used to predict personalized microbiome responses to dietary interventions and multi-omics and immune phenotyping data. These responses can be placed in a spatial context, thereby informing the rational development of nutritional interventions that can increase beneficial microbial metabolites [133,137].

## 9. Translational Perspectives for Nutritional Immunotherapy in Hepatocellular Carcinoma

Hepatocellular carcinoma (HCC) primarily occurs in chronically inflamed livers, where immune cells undergo functional reprogramming that drives a tumor-promoting microenvironment [138]. Clinical studies indicate that immune checkpoint inhibitors (ICIs) have shown promising efficacy in restoring anti-tumor immunity; the patient’s nutritional and inflammatory status affects their therapeutic efficacy [139,140]. This interplay marks the translational potential of nutritional immunotherapy as a complementary approach to improve clinical outcomes in HCC.

### 9.1. Concept of Nutritional Immunotherapy

Numerous studies have shown that changes in T cell metabolism can influence T cell differentiation and function [141,142]. Naïve T cells rely primarily on oxidative phosphorylation of lipids, amino acids, and glucose-derived pyruvate [142], while activated T cells necessitate a metabolic shift to support growth, proliferation, and cytokine production.

Metabolic degradation of amino acids like tryptophan and arginine generates important substrates for immune regulation [143]. Metabolism of arginine and methionine results in the synthesis of polyamines, regulating cell proliferation, nucleic acid stability, and they have been related to the initiation and regulation of inflammation and pathogen recognition. Fatty acids provide energy and structural components for cell membranes, and act as signaling molecules that modulate gene expression and T cell function [144,145]. They can also act as precursors for lipid mediators involved in immune responses and inflammatory pathways. In HCC, adequate nutritional status supports the immune microenvironment, T cell function is enhanced, and responsiveness to immune checkpoint inhibitors may be improved [146]. Nutritional immunotherapy focuses on the optimization of dietary intake of amino acids, fatty acids, vitamins, and other micronutrients to support immune function and potentially improve therapeutic responsiveness in patients with established cancer. Body mass index (BMI) and the prognostic nutritional index (PNI) are widely used.

### 9.2. Biomarkers and Diagnostics

Malnutrition and immunological status are closely linked to survival across various immune–nutritional indicators associated with postoperative outcomes and overall survival in HCC. The PNI, estimated using serum albumin levels and total peripheral blood lymphocyte counts, reveals both nutritional reserves and immune competence and has been extensively used to assess immune nutritional status. A tight relationship between PNI, tumorigenesis, and cancer progression in several malignancies, including small-cell lung, gastric, colorectal, breast cancer, and HCC [47,147].

The prognostic value of PNI in HCC was first reported by Pinato et al., who confirmed its independence as a predictor of overall survival. Subsequent clinical evidence suggests that the prognostic nutritional index (PNI) is significantly related to overall survival and disease-free survival in patients with very early and early-stage HCC undergoing curative surgical resection [148]. Subsequently, these findings raise the hypothesis that malnutrition and impaired immune status may indicate the reliability of survival in patients undergoing curative treatment for HCC.

Beyond biochemical indices, increasing attention has been directed toward the objective assessment of malnutrition and sarcopenia using imaging-based techniques. Recent evidence highlights the growing clinical relevance of sarcopenia in liver cancer, where computed tomography (CT)-based evaluation of skeletal muscle mass provides an accurate and objective measure of body composition. CT-derived assessment of skeletal muscle enables reliable identification of sarcopenia, which has been increasingly recognized as a significant prognostic factor in patients with hepatocellular carcinoma. Importantly, sarcopenia reflects not only nutritional depletion but also disease severity and systemic inflammation, offering prognostic information beyond traditional indices such as body mass index. Consequently, imaging-based muscle assessment complements conventional nutritional biomarkers and improves risk stratification and prognostic evaluation in liver cancer patients [18].

Moreover, immunological profiling developments have revealed the critical role of the tumor immune microenvironment (TME) in HCC prognosis and therapeutic response. The balance between immune effector cells and immunosuppressive populations within the TME is known to define distinct HCC subtypes with different clinical outcomes [149,150]. This understanding has been redefined by single-cell transcriptomic research, revealing the heterogeneity and functional states of immune cells within HCC tissues, pointing to the importance of integrated biomarker approaches that combine nutritional, immunological, and imaging parameters to guide precision treatment strategies.

### 9.3. Synergy with Immunotherapy

Clinical trials have reported objective response rates (ORRs) ranging from 15 to 20% in multiple single-agent studies of anti-CTLA-4 or anti-PD-1/PD-L1 inhibitors [150]. Because single-agent ICIs show limited ability to improve overall survival (OS), the combination of atezolizumab and bevacizumab has been suggested and tested in clinical trials [151]. These results show the need for synergistic approaches that enhance immunotherapy efficacy in HCC. Drawing on this evidence, combining immunotherapy with other strategies may represent an effective approach for treating HCC, as complex mechanisms and determinants of therapeutic efficacy underlie ICI resistance, including metabolic reprogramming of the HCC tumor microenvironment [152,153,154]. Preclinical studies indicate that metabolic reshaping of the HCC tumor microenvironment affects immune cell function, where nutrient deprivation and accumulation of metabolic intermediates impair anti-tumor immunity [154].

Preclinical models suggest that hypoxia due to rapid oxygen consumption and abnormal angiogenesis activates HIF and upregulates PD-L1 expression in the HCC tumor microenvironment [155]. As a result, lactate suppresses T-cell IFN-γ production, paving the way for T-cell exhaustion via the PD-1/PD-L1 pathway. By blocking the lactate transporter MCT-1, abundant lactate impedes the anti-tumor effects of cytotoxic T lymphocytes (CTLs) [156].

Experimental evidence demonstrates that the release of cytotoxic granzymes and perforin from CD8^+^ T-cells is inhibited by regulatory T-cells (Tregs). Additionally, lactate accumulation improves IL-6- and GM-CSF-regulated myeloid-derived suppressor cell (MDSC) production [33]. Overall, these mechanisms explain how metabolic reprogramming within the HCC TME contributes to immune evasion. These findings support the rationale for further investigation of combined ICIs and metabolic or nutritional interventions as adjunctive therapeutic strategies in established HCC. Representative clinical and translational studies linking nutritional status, microbiota-related factors, immunometabolic biomarkers, and therapeutic responsiveness in HCC are summarized in Table 4.

## 10. Conclusions

Diet-derived microbial metabolites such as SCFAs, bile acids, and tryptophan derivatives, as well as emerging metabolites including purines, polyphenols, and vitamins, contribute to the nutritional programming of the immune metabolome in HCC. These metabolites may boost antitumor immunity and increase the effectiveness of ICIs, but they can also encourage immunosuppression and resistance, demonstrating a “double-edged sword” effect. Metabolite-immune interactions are therefore essential to understanding the pathophysiology of liver cancer and offer new opportunities for improving immunotherapy tactics. These substances, produced by gut bacteria from food ingredients, can either increase resistance (kynurenine triggering immunosuppression, for example) or improve anti-tumor immunity (butyrate enhancing T-cell function). Prebiotics, postbiotics, and dietary interventions seek to modify this axis, offering a promising strategy to enhance immunotherapy efficacy; however, a better understanding of context-dependent effects and inter-individual variability is necessary.

## 11. Future Clinical Directions

1. Nutritional modulation based on mechanistic understanding should replace non-specific dietary recommendations, and interventions should be designed to influence major immunometabolic circuits such as AMPK-mTOR, FXR/TGR5, AhR, and redox-epigenetic signals in HCC.
2. Nutritional conditioning of antitumor immunity represents a promising strategy to improve antitumor immune responsiveness to immune checkpoint inhibitors by promoting mitochondrial metal homeostasis, improving effector T-cell metabolic performance, and inhibiting immunosuppressive pathways in the tumor microenvironment.
3. Gut microbiota composition, circulating microbial metabolites, and host immunometabolic profiles can inform precision nutrition approaches that stratify patients and enable tailored dietary interventions according to disease stage and etiology.
4. Diet-derived microbial metabolites, including short-chain fatty acids, bile acid derivatives, and tryptophan catabolites, have the potential to be non-invasive biomarkers for measuring immune competence, disease progression, and therapeutic response.
5. Hepatic immune and metabolic zonation should be considered as part of spatially informed intervention strategies, as the nutritional modulation of immune-excluded or metabolically dysregulated tumor niches may be feasible.
6. Integrative models based on artificial intelligence can support adaptive nutrition interventions, which can be achieved by combining dietary, microbial, metabolomic, and immune data, and optimizing therapeutic interventions dynamically.
7. Clinical trials need to be redesigned to consider nutrition and microbiota modulation as active therapeutic variables in order to demonstrate causality and accelerate clinical translation.

## Figures and Tables

**Table 1:** Nutritional components influencing the gut–liver axis in hepatic immunity.

Nutrient	Microbial Interaction	Key Metabolites	Effects on Hepatic Immunity	Key References
Omega-3 fatty acids	Enhances Akkermansia and Lactobacillus abundance and supports anti-inflammatory microbial profiles	Resolvins, protectins	Promotes anti-inflammatory macrophage polarization; reduces IL-6 production	[15]
Dietary fiber	Promotes SCFA-producing bacteria and increases microbial fermentation	Butyrate, acetate	Induces Treg differentiation; inhibits NF-κB activation	[9]
Vitamin D	Strengthens gut barrier integrity and modulates gut microbial composition	25(OH)D	Reduces endotoxemia; enhances T cell tolerance	[5]
Polyphenols	Enriches beneficial microbes and promotes microbial biotransformation	Phenolic acids	Activates Nrf2 signaling; inhibits NF-κB-mediated inflammation	[17]
Selenium	Regulates oxidative stress responses and microbial redox balance	Selenoproteins	Modulates macrophage antioxidant responses	[15]
Branched-chain amino acids (BCAAs)	Alters microbial fermentation and amino acid metabolism	Indole derivatives	Enhances mitochondrial oxidation; modulates AhR activity	[18]

**Table 2:** Diet-derived microbial metabolites and their immunometabolic effects in HCC.

Metabolite Class	Dietary Source	Microbial Pathway	Immune Modulation in HCC	Mechanistic Target	Key References
Short-chain fatty acids (SCFAs; acetate, propionate, butyrate)	Dietary fiber and complex polysaccharides	Microbial fermentation by Firmicutes and Bacteroidetes	Modulate hepatic immune homeostasis through effects on regulatory T-cell balance, inflammatory signaling, and hepatic stellate cell activation	GPR41, GPR43, GPR109A; HDAC inhibition; AMPK activation	[9,10,15,20]
Secondary bile acids (DCA, LCA)	Dietary fat and cholesterol	Bile salt hydrolase-mediated deconjugation and microbial dehydroxylation	Regulate macrophage activity and metabolic homeostasis; chronic exposure induces oxidative stress, DNA damage, and pro-tumorigenic signaling	FXR; TGR5	[5,11,14,19,21]
Tryptophan-derived indole metabolites (IPA, IAld, IAA)	Protein-rich foods and legumes	Indole production by *Lactobacillus*, *Bacteroides*, and *Clostridium* species	Promote intestinal barrier integrity, induce IL-22 signaling, and reduce hepatic inflammation; dysregulated metabolism contributes to immune suppression in the tumor microenvironment	AhR; IDO1	[4,7,16,18]
Polyphenol-derived metabolites (urolithins, phenolic acids)	Fruits, vegetables, tea	Microbial biotransformation by *Eubacterium ramulus* and *Clostridium* species	Activate antioxidant pathways, enhance cytotoxic T-cell activity, and suppress inflammatory signaling	Nrf2; SIRT1	[3,6,10,17]
Lipid-derived microbial metabolites (CLA, oxylipins)	Polyunsaturated fatty acid-rich foods	Fatty-acid modification by *Lactobacillus* species	Regulate macrophage polarization and induce apoptosis in hepatocytes, contributing to immunometabolic reprogramming	PPAR-γ	[14,19]

**Table 3:** Preclinical studies evaluating nutritional or microbiota-based modulation in experimental hepatocellular carcinoma (HCC) models.

Intervention/Model	Experimental Model	Main Mechanism	Immunometabolic Effect	Outcome	Reference
Probiotic supplementation (*Lactobacillus*, *Bifidobacterium*, *Akkermansia muciniphila*)	Experimental HCC models	Restoration of gut microbial balance and reduced LPS translocation	Reduced TLR4-mediated inflammation and improved immune regulation	Suppressed tumor-promoting inflammatory signaling	[34,69]
SCFA-producing microbiota/butyrate	Murine HCC models	HDAC inhibition and metabolic reprogramming	Enhanced CD8^+^ T-cell activity and anti-inflammatory macrophage polarization	Improved antitumor immune responses	[34,104]
Acetate-mediated immune modulation	Experimental HCC and PD-1 blockade models	Suppression of IL-17A signaling	Reduced immunosuppressive signaling and improved cytotoxic T-cell infiltration	Enhanced responsiveness to immunotherapy	[105]
Prebiotic supplementation (inulin, resistant starch, fructo-oligosaccharides)	Experimental liver disease/HCC models	Increased SCFA production and epithelial barrier integrity	Reduced systemic inflammation and microbial translocation	Delayed inflammatory progression associated with hepatocarcinogenesis	[107]
Polyphenol-rich dietary interventions	Experimental HCC models	Activation of Nrf2 signaling and suppression of NF-κB pathways	Reduced oxidative stress and inflammatory macrophage activation	Attenuated tumor-promoting inflammatory signaling	[113,115]
Omega-3 polyunsaturated fatty acids (PUFAs)	Experimental HCC models	Modulation of COX-2/PGE2 signaling and fatty acid oxidation	Enhanced CD8^+^ T-cell metabolic function and reduced inflammatory signaling	Improved antitumor immune microenvironment	[118]
Caloric restriction/intermittent fasting	Experimental HCC models	AMPK activation and mTOR inhibition	Reduced MDSC proliferation and enhanced immune stress resistance	Improved immunometabolic regulation	[127]

**Table 4:** Clinical studies linking nutrition, microbiota-related factors, biomarkers, and immunotherapy responsiveness in hepatocellular carcinoma (HCC).

Study Type/Population	Nutritional or Microbiota-Related Variable	Biomarker/Outcome	Main Finding	Clinical Implication	Reference
HCC patients undergoing curative resection	Prognostic Nutritional Index (PNI)	Overall survival (OS), disease-free survival (DFS)	Lower PNI was associated with poorer survival outcomes	Nutritional and immune status may influence prognosis in HCC	[148]
Patients with advanced HCC	Sarcopenia assessed by CT imaging	Survival and treatment outcome	Sarcopenia correlated with disease severity and reduced survival	Imaging-based nutritional assessment may improve risk stratification	[97]
HCC patients receiving immunotherapy	Immune-metabolic tumor microenvironment	ICI responsiveness	Metabolic dysregulation and immune exhaustion were linked to reduced immunotherapy response	Nutritional/metabolic interventions may improve ICI efficacy	[139,140]
Patients with chronic liver disease/HCC	Gut microbiota diversity and SCFA production	Inflammation and immune regulation	Reduced microbial diversity and altered SCFA production were associated with inflammatory progression	Microbiota-targeted nutritional approaches may support immune homeostasis	[110]
Clinical/translational studies in HCC	Mediterranean-style dietary patterns	Systemic inflammation and metabolic regulation	Anti-inflammatory dietary patterns were associated with improved metabolic and immune profiles	Diet composition may support prevention and adjunctive therapeutic strategies	[119]
HCC patients receiving combined therapies	Nutritional status and immune biomarkers	Immunotherapy responsiveness	Nutritional and immune parameters may predict treatment responsiveness	Precision nutrition approaches may support personalized HCC management	[30,157]

## Data Availability

No new data were created or analyzed in this study.

## Abbreviations

The following abbreviations are used in this manuscript:

ACOT12	Acetyl-CoA thioesterase 12
ACSS2	Acyl-CoA synthetase short-chain family member 2
AhR	Aryl hydrocarbon receptor
AMPK	AMP-activated protein kinase
ARNT	AhR nuclear translocator
BMI	Body mass index
BTB	Broad-complex, Tramtrack and Bric-à-brac domain
CAF	Cancer-associated fibroblast
CD8^+^	Cluster of differentiation 8-positive T cell
CT	Computed tomography
CTL	Cytotoxic T lymphocyte
CTLA-4	Cytotoxic T-lymphocyte-associated protein 4
CXCL11	C-X-C motif chemokine ligand 11
CYP1A1	Cytochrome P450 family 1 subfamily A member 1
CYP1A2	Cytochrome P450 family 1 subfamily A member 2
CYP1B1	Cytochrome P450 family 1 subfamily B member 1
CYP8B1	Cholesterol 12α-hydroxylase
DCA	Deoxycholic acid
DFS	Disease-free survival
DLG	Asp-Leu-Gly motif
DNA	Deoxyribonucleic acid
ER	Endoplasmic reticulum
ETGE	Glu-Thr-Gly-Glu motif
FXR	Farnesoid X receptor
GEMs	Genome-scale metabolic models
GM-CSF	Granulocyte-macrophage colony-stimulating factor
H3K9Ac	Histone H3 lysine 9 acetylation
HCC	Hepatocellular carcinoma
HDAC	Histone deacetylase
HIF	Hypoxia-inducible factor
IDO1	Indoleamine 2,3-dioxygenase 1
IFN-γ	Interferon gamma
IL-17A	Interleukin 17A
IL-22	Interleukin 22
IMC	Imaging Mass Cytometry
ICI	Immune checkpoint inhibitor
ILC3	Type 3 innate lymphoid cell
IVR	Intervening region
Keap1	Kelch-like ECH-associated protein 1
LPS	Lipopolysaccharide
MASLD	Metabolically-dysfunction-associated steatotic liver disease
MAT1A	Methionine adenosyltransferase 1A
MAT2A	Methionine adenosyltransferase 2A
MCT-1	Monocarboxylate transporter 1
MDSC	Myeloid-derived suppressor cell
MLCK	Myosin light chain kinase
mRNA	Messenger RNA
mTOR	Mechanistic target of rapamycin
NADPH	Nicotinamide adenine dinucleotide phosphate
NF-κB	Nuclear factor kappa B
NK	Natural killer
NOX	NADPH oxidase
Nrf2	Nuclear factor erythroid 2-related factor 2
ORR	Objective response rate
OS	Overall survival
PAS	Per-ARNT-Sim
PD-1	Programmed cell death protein 1
PD-L1	Programmed death-ligand 1
PNI	Prognostic nutritional index
PGE2	Prostaglandin E2
PUFA	Polyunsaturated fatty acid
RBX1	RING-box protein 1
ROS	Reactive oxygen species
SAM	S-adenosyl-L-methionine
SCFA	Short-chain fatty acid
SIRT3	Sirtuin 3
SPM	Specialized pro-resolving mediator
TGR5	Takeda G protein-coupled receptor 5
TLR4	Toll-like receptor 4
TME	Tumor microenvironment
Treg	Regulatory T cell
XRE	Xenobiotic response element
